# Overexpression of lipocalin 2 in human cervical cancer enhances tumor invasion

**DOI:** 10.18632/oncotarget.7096

**Published:** 2016-01-31

**Authors:** I-Hsiao Chung, Tzu-I Wu, Chia-Jung Liao, Jin-Yo Hu, Yang-Hsiang Lin, Pei-ju Tai, Chyong-Huey Lai, Kwang-Huei Lin

**Affiliations:** ^1^ Department of Biochemistry, School of Medicine, Chang-Gung University and Liver Research Center, Chang Gung Memorial Hospital, Taoyuan, Taiwan 333; ^2^ Department of Obstetrics and Gynecology, Chang Gung Memorial Hospital and Chang Gung University College of Medicine, Taoyuan, Taiwan 333; ^3^ Department of Obstetrics and Gynecology, Wan Fang Hospital, Taipei Medical University, Taipei, Taiwan 116; ^4^ Gynecologic Cancer Research Center, Chang Gung Memorial Hospital, Taoyuan, Taiwan 333

**Keywords:** lipocalin2, cervical cancer, invasion, metastasis

## Abstract

Cervical carcinoma is the third-most common cause of cancer-related deaths in women worldwide. However, the molecular mechanisms underlying the metastasis of cervical cancer are still unclear. Oligonucleotide microarrays coupled with bioinformatics analysis show that cytoskeletal remodeling and epithelial-to- mesenchymal transition (EMT) are significant pathways in clinical specimens of cervical cancer. In accord with clinical observations demonstrating ectopic expression of lipocalin 2 (LCN2), an oncogenic protein associated with EMT, in malignant tumors, was significantly upregulated in cervical cancer and correlated with lymph node metastasis. Overexpression of LCN2 enhanced tumor cell migration and invasion both *in vitro* and *in vivo*. Conversely, knockdown or neutralization of LCN2 reduced tumor cell migration and invasion. LCN2-induced migration was stimulated by activation of the EMT-associated proteins, Snail, Twist, N-cadherin, fibronectin, and MMP-9. Our findings collectively support a potential role of LCN2 in cancer cell invasion through the EMT pathway and suggest that LCN2 could be effectively utilized as a lymph node metastasis marker in cervical cancer.

## INTRODUCTION

Cervical cancer is the third leading cause of cancer-related mortality among women worldwide [1]. Surgery has long been a standard therapy for early-stage (I–IIa) disease, but limitations in the diagnosis of early lymph node metastasis of invasive cervical cancer remain [2]. The available serum tumor markers, such as squamous cell carcinoma antigen and carcinoembryonic antigen, are elevated in 30%–40% of patients with early-stage disease and in approximately 70% of patients with late-stage disease, but neither are specific diagnostic or prognostic factors [3]. Surgical treatment does not help patients with metastatic disease, and the outcome of radiotherapy is based on the detection of metastasis at diagnosis [4]. Hence, markers of early metastasis would be helpful in selecting the treatment modality. However, there are currently no available markers for early metastasis or indicators of successful treatment. Therefore, diagnostic or prognostic markers for carcinoma of the uterine cervix are critically important because of their potential value in basic research efforts to understand the characteristics of the tumor, as well as clinical management strategies to improve patient survival and quality of life.

Persistent infection with human papillomavirus (HPV) is a major risk factor for both cervical squamous cell carcinoma (SCC) and cervical adenocarcinoma (AD) [5]. However, HPV alone is not sufficient to cause cervical metastasis [6]; other molecular factors and cervical carcinogenesis pathways are essential. Our oligonucleotide microarrays revealed that the oncogenic protein, lipocalin 2 (LCN2), is overexpressed in clinical specimens of cervical cancer. LCN2, a 24 kDa, secreted glycoprotein, shares structural and functional similarities with other members of the lipocalin family, including eight anti-parallel β-sheets that form a cup-shaped ligand-binding site for small lipophilic molecules and a role as a transporter in a normal physiological environment [7]. Recent studies have reported increased LCN2 expression in various human pathologies, including kidney diseases [8] and obesity [9], as well as solid tumors [10, 11] and other cancers, such as leukemia [12], breast cancer [13], intrahepatic cholangiocarcinoma [14] and liver cancer [15]. Several possible molecular mechanisms underlying LCN2 functions in tumor progression have been demonstrated, including promotion of epithelial to mesenchymal transition (EMT) and modulation of matrix metallopeptidase (MMP)-9 activity [15]. Hallmarks of EMT include loss of the epithelial marker E-cadherin, an increase in the mesenchymal markers vimentin, N-cadherin and fibronectin, and an increase in migratory and invasive behavior [16]. Notably, the mechanisms underlying LCN2 actions in cervical cancer are still unclear. Here, we investigated the role of LCN2 in cervical cancer, focusing on the mechanism responsible for metastasis.

## RESULTS

### High expression of LCN2 in cervical cancer is correlated with tumor metastasis

**Figure 1:**
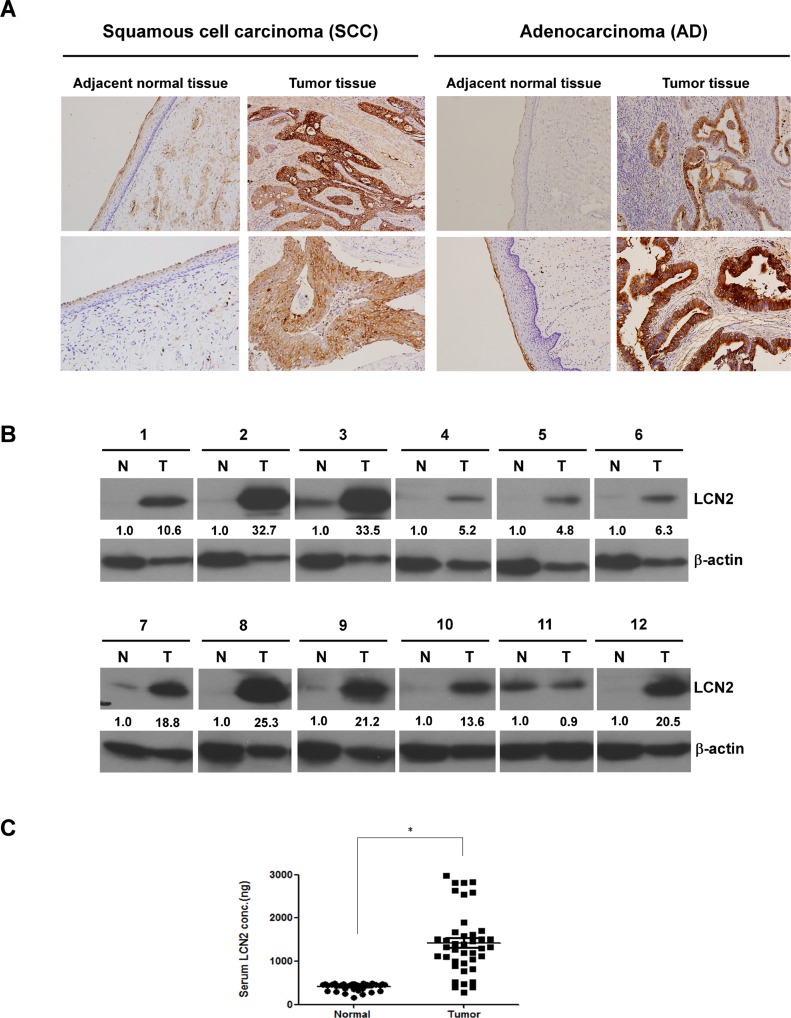
LCN2 is up-regulated in clinical specimens of cervical cancer (**A**) LCN2 protein levels determined by IHC in two paired sections from SCC (left panel) and AD (right panel) patients. (**B**) LCN2 expression was detected by Western blotting in 12 representative cervical cancer tissues. (**C**) Serum LCN2 protein levels were determined by enzyme-linked immunosorbent assays (ELISAs) in 37 healthy donors and 40 cervical cancer patients.

To verify the overexpression of LCN2 in cervical cancer, we performed immunohistochemistry (IHC) on 90 clinical specimens, including those from SCC (*n* = 43), AD (*n* = 28), and adenosquamous cell carcinoma (ADSCC) (*n* = 19) patients. The mean score in tumor tissues for all 90 samples (111.31 ± 73.76) was significantly greater than that in the matching adjacent tissue (9.85 ± 26.13; *P* < 0.001). To determine the clinicopathological significance of LCN2 expression, we analyzed the IHC scores for LCN2. Notably, among SCC patients, LCN2 was significantly increased in patients with higher FIGO stage (*P* < 0.033), poorly differentiated grade (*P* = 0.042) and lymph node involvement (*P* = 0.044) (Table 1). On the contrary, LCN2 expression was reduced in AD patients with lymph node involvement in comparison with those without lymph node metastasis (*P* < 0.009), and similar expression level in ADSCC patients irrespective of lymph node involvement (Table S1). LCN2 staining was most strongly detected in two pairs of SCC (Figure 1A, left panel) and AD (Figure 1A, right panel), where it was found in the cytosol of cervical cancer cells. Increased LCN2 expression in cervical cancer tissues of 12 representative paired specimens is shown in Figure 1B. Serum levels of LCN2 were also significantly increased (∼5 to 6-fold) in 40 patients compared to 37 healthy donors (Figure 1C). Collectively, these results support the association between LCN2 expression and lymph node metastasis, and suggest that LCN2 is a potential diagnostic marker for cervical cancer.

**Table 1:** Clinicopathologic correlations of LCN2 expression in patients with cervical cancer (Squamous cell carcinoma, SCC)

Parameter	*n*	LCN2 histoscore	*P* value
**Age:**			
≤ 50	7	68.57 ± 84.544	0.391
> 50	36	77.08 ± 55.605	
**FIGO stage:**			
I a1	1	50	0.090
I b1	33	66.21	
I b2	4	120	
II a	4	130	
II b	1	20	
**FIGO stage:**			
≤ I b1	34	65.74 ± 56.944	0.033*
> I b1	9	113.33 ± 59.582	
**Histological grade:**			
Well/Moderately	18	57 ± 49.59	0.042*
Poorly	23	94.13 ± 64.54	
**Tumor size(image):**			
≤ 4 cm	25	70.2 ± 49.212	0.374
> 4 cm	4	115 ± 88.129	
**Depth of penetration:**			
≤ 50%	17	81.76 ± 70.466	1.00
> 50%	16	71.56 ± 48.087	
**Lymph node involvement:**			
No	35	68.71 ± 58.944	0.044*
Yes	7	118.57 ± 51.455	

### Altered EMT signaling pathway components in clinical specimens of cervical cancer

An Affymetrix microarray was used to identify gene expression patterns in cervical cancer specimens. An analysis of differentially expressed genes using the MetaCore pathway analysis tool revealed significant enrichment of 10 pathway maps (Table S2). Notably, one cytoskeleton remodeling map (Table S2, number 1) and three EMT-related maps (Table S2, numbers 2, 8, and 9) were enriched. The cytoskeleton remodeling and EMT signaling pathways regulate cell migration and invasion, and promote cancer progression. The EMT signaling-related gene, LCN2, was up-regulated ∼21-fold in clinical specimens of cervical cancer, supporting our hypothesis that LCN2 modulates cervical cancer progression through the EMT pathway.

### LCN2 is associated with cervical cancer progression

**Figure 2:**
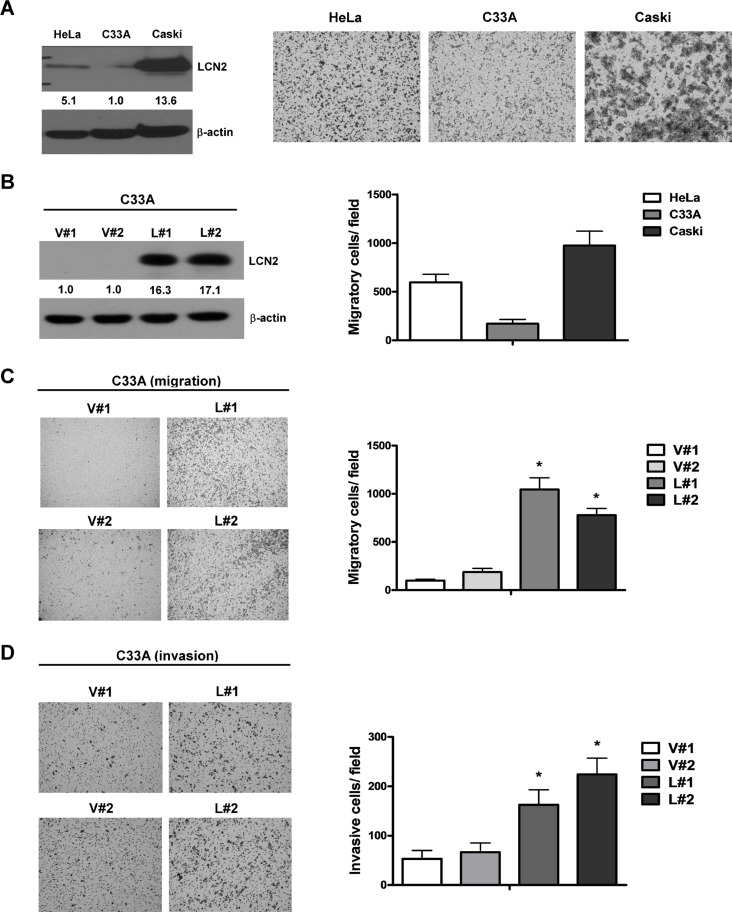
LCN2 promotes cervical cancer cell migration and invasion *in vitro* (**A**) *Left panel*: Endogenous LCN2 expression was analyzed in C33A, HeLa and Caski cervical cancer cell lines by Western blotting. *Right panel*: Migration ability was analyzed in the three cervical cancer cell lines using Transwell assays. Quantified data is shown below. (**B**) LCN2 expression in C33A cells was detected in LCN2-overexpressing clones (L#1, L#2) and controls (V#1, V#2) by Western blot analysis. (**C**) Migration ability was analyzed in LCN2 -overexpressing and control C33A cell lines using Transwell assays. The number of cells traversing the filter to the lower chamber was counted to determine migration activity. *Left panel*: Transwell filters stained with crystal violet. *Right panel*: Quantification of migration ability. (**D**) Invasion ability was examined using Transwell assays. Differences were analyzed using a Kruskal–Wallis test (*P < 0.05).

LCN2 expression was identified in three cervical cancer cell lines (Figure 2A, left panel). The expression levels of endogenous LCN2 protein were positively correlated with cell mobility (Figure 2A, right panel). To determine the specific functions of LCN2, we established LCN2-overexpressing sublines, C33A-L#1 and -L#2, and control cell lines, C33A-V#1 and -V#2 (Figure 2B, left panel). Notably, C33A cell lines overexpressing LCN2 displayed significantly increased migration (∼5–6-fold) and invasion (∼3–5-fold) compared with control cells (Figure 2C, 2D). These results indicate that LCN2 functions in cell migration and invasion.

### LCN2 depletion suppresses cervical cancer progression

**Figure 3:**
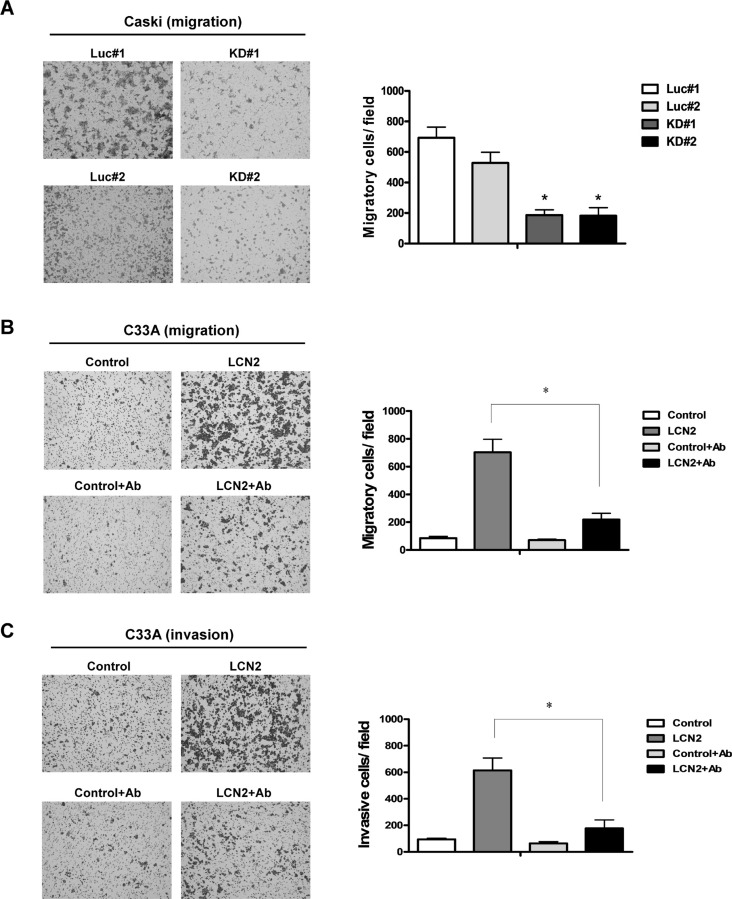
LCN2 depletion suppresses cervical cancer cell mobility *in vitro* (**A**) *Left panel*: Migration ability was analyzed in LCN2-depleted and control Caski cell lines using Transwell assays. *Right panel*: Quantification of migration assay results. (**B**) Application of the LCN2-neutralizing antibody in LCN2-overexpressing and vector control (pcDNA3.0) C33A cells. *Left panel*: Comparison with IgG antibody treatment. *Right panel*: Quantification of migration ability. (**C**) *Left panel*: Invasion abilities of C33A cells treated with the LCN2 -neutralizing antibody. *Right panel*: Quantification of invasion ability. Differences were analyzed using a Kruskal–Wallis test (*P < 0.05).

To determine the consequences of LCN2 depletion, we established LCN2-knockdown lines, Caski-KD#1 and -KD#2, and control cell lines, Caski-Luc#1 and Luc#2 (Figure 3A, left panel). Transwell assays showed that depletion of LCN2 in Caski cells decreased migration ability compared with control cells (Figure 3A, right panel). Consistent with this, treatment with an LCN2-neutralizing antibody also significantly decreased migration (∼3-fold) and invasion (∼3.5-fold) in C33A-LCN2 overexpressing cells, but not in control cells (Figure 3B, 3C). Taken together, these data confirm the ability of LCN2 to accelerate tumor cell migration.

### LCN2 is associated with cervical cancer progression *in vivo*

**Figure 4:**
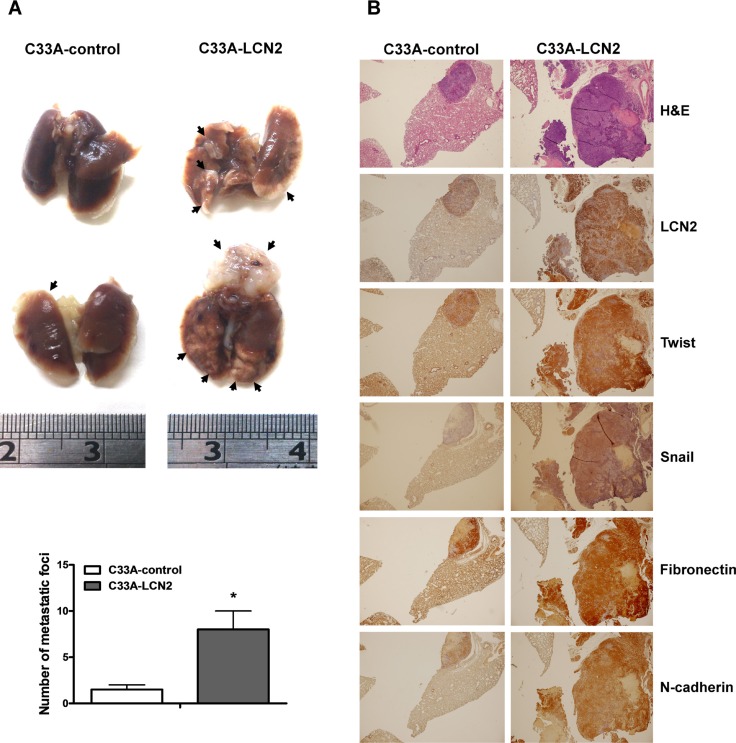
LCN2 promotes cervical cancer cell migration and invasion *in vivo* (**A**) *Upper panel*: Images depicting lung tumor foci of C33A-control and C33A-LCN2 cells. *Lower panel*: Quantification of metastatic foci in lungs. (**B**) *Upper panel*: Tumor foci in C33A-control and C33A-LCN2 cells examined using H & E staining. *Lower panel:* LCN2, Snail, Twist, N-cadherin and fibronectin protein levels in C33A-control and C33A-LCN2 cells determined by IHC. Differences were analyzed using a Mann–Whitney *U* test (*P < 0.05).

To verify that the *in vitro* effects of LCN2 can be replicated *in vivo*, we created a xenograft tumor model by injecting SCID mice with C33A-LCN2 overexpressing or C33A-control cells. Notably, C33A-LCN2 cells formed significantly higher numbers of lung foci in SCID mice (Figure 4A) compared with control cells, and displayed elevated LCN2 expression, as evidenced by H & E staining and IHC staining for LCN2, respectively (Figure 4B, upper panel). The EMT-associated markers Snail, Twist, N-cadherin, and fibronectin were similarly increased in serial sections of mice specimens (Figure 4B). Thus, LCN2 appears to promote cell migration and invasion in C33A cervical cancer cells, both *in vitro* and *in vivo*.

### LCN2 regulates EMT-related proteins in cervical cancer cell

**Figure 5:**
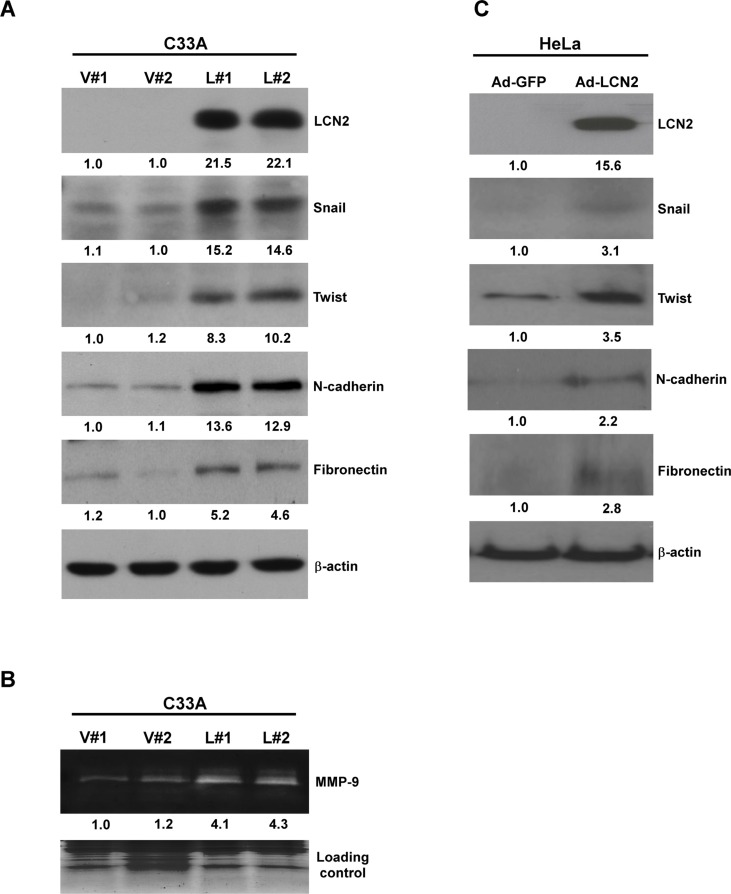
LCN2 regulates cell mobility through the EMT pathway Expression of Snail, Twist, N-cadherin and fibronectin (**A**), MMP-9 activity (**B**), and Western blot determination of LCN2 expression levels following adenovirus infection in LCN2-overexpressing (L#1, L#2) and control (V#1, V#2) C33A cells.

Pathway analysis and animal results indicate that LCN2 may promote cancer cell migration and invasion through EMT. Accordingly, we examined whether the EMT pathway is involved in the LCN2-induced phenotypes. Marked upregulation of Snail, Twist, N-cadherin, fibronectin, and MMP-9 were observed in LCN2-overexpressing cells (C33A-LCN2, L#1 and L#2) compared with control cells (C33A-control, V#1 and V#2) (Figure 5A, 5B). In a HeLa cell model, cells with adenoviral-mediated overexpression of LCN2 (Ad-LCN2) exhibited higher levels of Snail, Twist, N-cadherin, and fibronectin expression or equal fluorescence intensity compared with control cells (Ad-GFP) (Figure 5C, S1).

### LCN2 promotes cervical cancer cell metastasis via the EMT pathway

**Figure 6:**
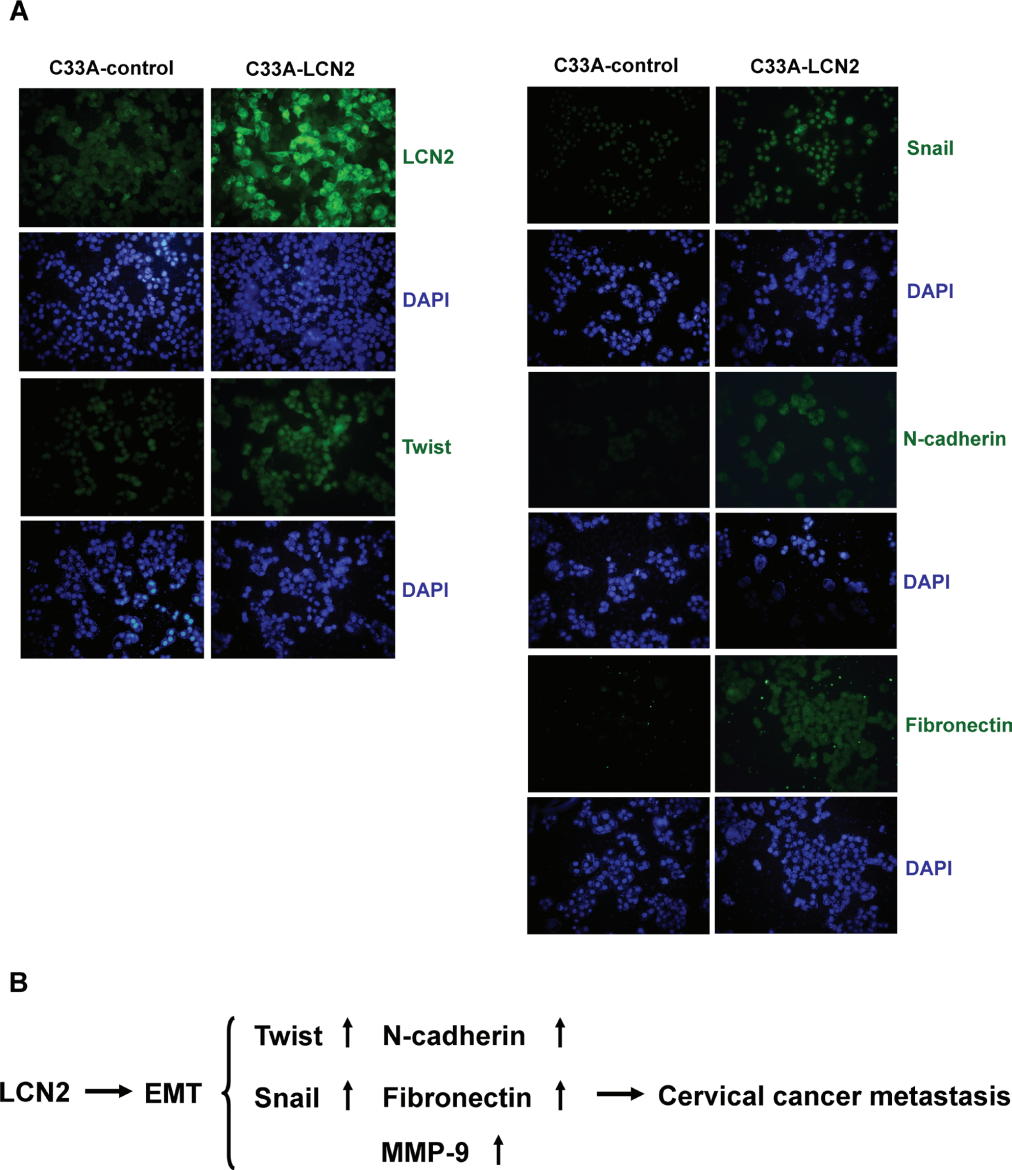
Expression of EMT markers in LCN2-overexpressing cervical cancer cell (**A**) Immunofluorescence detection of LCN2, Snail, Twist, N-cadherin and fibronectin expression in LCN2-overexpressing and control C33A cells. (**B**) Schematic depiction of LCN2 promotion of cervical cancer cell metastasis and cancer progression through EMT by modulation of the EMT markers Snail, Twist, N-cadherin, and fibronectin.

To further confirm the involvement of the EMT pathway in LCN2-induced phenotypes, we used immunofluorescence staining to identify the expression and location of EMT-related proteins in cervical cancer cells. These experiments revealed marked upregulation of Snail and Twist in the nucleus, and N-cadherin and fibronectin in the cytoplasm of LCN2-overexpressing cells (C33A-LCN2) compared with control cells (C33A-control) (Figure 6A). Collectively, these results support a potential role of LCN2 in cervical cancer metastasis by promoting cancer cell migration and invasion through modulation of the EMT pathway components, Snail, Twist, N-cadherin, and fibronectin (Figure 6B).

## DISCUSSION

The molecular mechanisms involved in the metastasis of cervical cancer are still unclear. To identify the pathways underlying cervical carcinogenesis, *oligonucleotide* microarray coupled with a bioinformatics analysis were used to identify genes differentially expressed in cervical cancer. Further, clinicopathological parameters and the diagnostic significance of the aberrantly expressed proteins were evaluated to identify potential biomarkers of cervical cancer metastasis. LCN2 is highly expressed in cervical tumors (SCC, AD and ADSCC) compared with adjacent normal tissues. In the cervical cancer patients examined, elevated expression of LCN2 was significantly associated with lymph node involvement in patients with SCC. Nevertheless, LCN2 overexpression and lymph node involvement were inversely correlated in patients with AD, and no differential expression of LCN2 in patients with ADSCC irrespective of lymph node metastasis. The observational clinical result might be explained the diverse effect of LCN2 in EMT carcinogenesis pathway between SCC and AD/ADSCC, thus more detailed molecular mechanism and larger study samples to elucidate the role of LCN2 are warranted. In cases where the secretory component of LCN2 is easily released into the circulation, elevated serum LCN2 levels was associated with distant metastases. Thus, serum LCN2 may be useful as a circulating biomarker for cervical cancer distant metastases.

In the current study, LCN2 is up-regulated at the protein level in clinical specimens. In addition, cell lines overexpressing LCN2 showed higher migration and invasion abilities, both *in vitro* and *in vivo*. Moreover, LCN2 enhanced cell invasion via the EMT pathway, leading to cancer cell progression. Overexpression of LCN2 promoted a mesenchymal-like cell morphology accompanied by increased expression of mesenchymal markers (Snail, Twist, N-cadherin, fibronectin and MMP9) that contribute to invasiveness, which accounts for their role in enhancing tumor cell motility in metastasis [25, 26]. LCN2 has been shown to regulate the HIF-1α (hypoxia-inducible factor 1α)/VEGF (vascular endothelial growth factor) cascade through activation of ERK (extracellular signal-regulated kinase) and enhancement of angiogenesis in the aggressive MDA-MB-231 cell line [27]. Knockdown of LCN2 suppresses the invasion of prostate cancer cells through downregulation of MMP-9 [28]. In breast cancer, LCN2 is stimulated by the HER2 (human epidermal growth factor receptor 2)/PI3K (phosphoinositide 3-kinase)/AKT pathway [29]. Conversely, a decrease in LCN2 expression significantly reduces the invasion and migration abilities of HER2-positive breast cancer cells [30].

LCN2 is overexpressed in the intestine in colitis patients and acts as a negative prognostic indicator in colorectal cancer [31]. However, several studies have reported that LCN2 suppresses cell migration and invasion in colon cancer and in Ras-transformed mouse mammary cells [32]. LCN2 has also been demonstrated to inhibit invasion and angiogenesis in pancreatic cancer [33]. In a study by Wang and colleagues [34], knockdown of nm23-H1 in SiHa uterine cervical cancer cells was shown to increase LCN2 promoter activity and gene expression, thereby decreasing cell migration and invasion. These authors' investigation of the correlation between LCN2 and nm23-H1 expression in cancer metastasis suggested that the *LCN2* gene is an important downstream target of nm23-H1 in SiHa cancer cells. However, the regulatory pathway identified and cell types investigated by these authors are different from those in the current study. Moreover, their clinicopathological data do not fully support a suppressive role of LCN2. In fact, LCN2 expression is higher in cervical carcinoma compared to high- and low-grade dysplasia tissue [35]. In addition, overexpression of LCN2 in C33A cells led to an increase in Snail, Twist, N-cadherin and fibronectin protein levels as well as MMP-9 activity. The mesenchymal markers Snail and Twist are core transcription factors in the EMT process, and stimulate the downstream target genes encoding N-cadherin, fibronectin and MMP-9 [36, 37]. Our findings consistent with the conclusion that activation of these mesenchymal makers leads to cervical cancer cell metastasis. In conclusion, LCN2 enhances migration and invasion abilities in cervical cancer cell lines, both *in vitro* and *in vivo*. LCN2 may stimulate cervical cancer cell metastasis by promoting cancer cell motility through activation of EMT pathway components.

## MATERIALS AND METHODS

### Patients

A total of 90 patients with cervical carcinoma to underwent primary definitive surgery between 2000 and 2008 at Chang Gung Memorial Hospital, Taoyuan, Taiwan, was retrieved from the hospital database and was enrolled for prognosis analysis under the Institutional Review Board-approved protocol (IRB: 95-1241B) with the informed consent. The patients, comprising stage I to IIB disease, were clinically staged according to the International Federation of Gynecology and Obstetrics (FIGO) staging system. The fresh clinical specimens and paraffin-embedded tissues of the 90 patients were obtained from hospital archive. The patient and tumor characteristics were listed in Table 1. The fresh tumor and paired adjacent non-cancerous specimens from 65 of the 90 patients were available and used in Western blot analysis. The histological types of the 90 patients were SCC in 43 patients, AD in 28 patients and ADSCC in 19 patients. The available paraffin-embedded tissues from 90 cervical cancer patients were sectioned and used in the immunohistochemical (IHC) and statistical analyses. The median age of the 90 patients was 57 years-old (range, 35–84). The median follow-up time of the survived patients among 90 cases was 61 months (range, 6–114 months). There were 26 patients with relapse and 16 patients died during the follow-up period. Lymph node metastases were histologically confirmed in 26 (29.5%) of 88 patients to be aware of lymph node status.

### RNA extraction and affymetrix oligonucleotide microarrays

Total RNA from paired cervical squamous cell carcinoma and adjacent noncancerous mucosa were extracted using the TRIzol reagent (Life Technologies, Rockville, MD), as described previously [17, 18]. Total RNA (20 μg) was used for labeling and hybridization with the Affymetrix GeneChip Human genome U133A 2.0 array (Affymetrix, Santa Clara, CA) containing 14,500 human genes. The slides were scanned, and intensities were acquired using GenePix Pro 4.1 software (Axon Instruments Inc. Foster City, CA).

### Cell culture

C33A, HeLa, and Caski human cervical cell lines were routinely cultured at 37°C in a humidified atmosphere of 95% air and 5% CO_2_ in Dulbecco's modified Eagle's medium (DMEM) supplemented with 10% fetal bovine serum (FBS). The C33A cell line was stably transfected with LCN2 (C33A-V#1 and C33A-V#2).

### Preparation of conditioned medium

C33A-LCN2 (L#1 and L#2) and HeLa-LCN2 (Ad-LCN2) cells were grown to confluence in 10-cm cell culture dishes. Adherent cells were washed twice with phosphate-buffered saline (PBS) and subsequently incubated in serum-free medium. At the end of the culture period, conditioned medium was collected and concentrated using spin columns with a molecular mass cut-off of 3 kDa (Amicon Ultra, Millipore, Billerica, MA).

### Cloning of LCN2

cDNA was synthesized from total RNA (1 μg) using Superscript II reverse transcriptase (Invitrogen, Carlsbad, CA) and oligo (dT) primers. *LCN2* was amplified from cDNA by polymerase chain reaction (PCR) using the primer pair 5′-TCA GGT ACC ATG CCC CTA GGT CTC CTG TG-3′ (forward) and 5′-CTC CTC GAG TCA GCC GTC GAT ACA CTG GT-3′ (reverse), and the following thermocycling conditions: 30 cycles at 95°C for 1 min, 58°C for 1 min, and 72°C for 2 min. The *LCN2* open reading frame was ligated into the pcDNA 3.0 expression vector, and the resulting construct was sequenced to confirm the presence of the gene.

### Immunoblot analysis

Total cell lysates and conditioned media were prepared, and protein concentrations were determined using a Bradford assay kit (Pierce Biotechnology, Rockford, IL). Equivalent amounts of proteins were fractionated by sodium dodecyl sulfate-polyacrylamide gel electrophoresis (SDS-PAGE) on a 10% gel. Separated proteins were transferred to a nitrocellulose membrane (pH 7.9; Amersham Biosciences Inc., Piscataway, NJ), blocked with 5% non-fat powdered milk, and incubated with specific anti-LCN2 (R & D Systems; AF1757), anti-Twist (GeneTex; GTX127310), anti-Snail (Cell Signaling; #6615), anti-N-cadherin (Invitrogen; 33–3390) and anti-fibronectin (Santa Cruz Biotechnology; sc-9068) primary antibodies at 4°C overnight. After washing, membranes were incubated with HRP-conjugated anti-mouse, anti-rabbit or anti-goat IgG secondary antibody, as appropriate, for 1 h at room temperature. Immune complexes were visualized using an enhanced chemiluminescence (ECL) detection kit (Amersham) and Fuji X-ray film.

### Establishing C33A cell lines stably overexpressing LCN2

The C33A cell line, grown in 10-cm cell culture dishes, was transfected with the LCN2 expression plasmid using the Lipofectamine reagent (Invitrogen). After 24 h, transformants were selected from transfected cells by growing in medium containing the antibiotic G418 (400 μg/ml). Expression of LCN2 protein in the selected clones was detected using Western blot analysis.

### shRNA-mediated LCN2 knockdown

Short hairpin RNA (shRNA) sequences targeting LCN2 were purchased from the National RNAi Core Facility (Institute of Molecular Biology, Academia Sinica, Taiwan). The Caski cell line was transiently transfected with shRNA targeting the endogenous *LCN2* gene using the Turbofect reagent (Invitrogen). LCN2 repression was confirmed by Western blot analysis.

*In vitro* migration and invasion assays

The influence of LCN2 on the migration and invasion abilities of C33A-LCN2 cells was determined *in vitro* using Transwell assays (Falcon BD, Franklin Lakes, NJ), as described previously [19]. Briefly, cell density was adjusted to 10^5^ cells/ml, and 100 μl of the suspension was seeded on upper chambers of the Transwell plate, either coated (invasion) or not coated (migration) with Matrigel (Becton-Dickinson). For both assays, the pore size of the upper chamber was 8 mm. The medium in the upper chamber was serum-free DMEM, and the lower chamber contained DMEM supplemented with 20% fetal bovine serum (FBS), included as a chemoattractant. After incubation for 24 h at 37°C, cells traversing the filter from the upper to lower chamber were stained with crystal violet and counted. Experiments were repeated at least three times.

### Immunohistochemistry staining

Formalin-fixed and paraffin-embedded tissues from lungs of SCID mice were evaluated by hematoxylin and eosin (H & E) staining and immunohistochemistry using polyclonal antibodies against LCN2 (Epitomics; #3639–1), Snail (GeneTex; GTX125918), N-cadherin (GeneTex; GTX112734), and fibronectin (GeneTex; GTX112794) using the avidin-biotin complex method. Positive staining of cancer cells for LCN2 was identified by a dark brown color indicative of LCN2 immunoreactivity. The staining intensity was graded as absent (0), weak (1+), medium (2+), or strong (3+). The histoscore (Q) was calculated by multiplying the percentage (P) of positive cells by the intensity (I), according to the formula: Q = P × I. The mean Q of each type of cervical cancer was chosen as previously described [20, 21].

### Gelatin zymography

Supernatant fractions of C33A-LCN2 cells cultured for 24 h were collected and concentrated using Amicon Ultra-4 centrifugal filter devices (Merck Millipore Ltd). Equal amounts of proteins were separated by SDS-PAGE on 10% gels containing 0.1% gelatin (Sigma, St. Louis, MO). The gel was incubated in reaction buffer (40 mM Tris-HCl pH 8, 10 mM CaCl_2_, 1% NaN_3_) at 37°C overnight, stained with 0.25% Coomassie Brilliant Blue R-250 in 10% acetic acid and 50% methanol for 30 min, and de-stained twice with 10% acetic acid and 20% methanol for 30 min each.

### Immunofluorescence staining

Cells were seeded on glass slides, fixed with 3.7% paraformaldehyde, permeabilized with 0.1% Triton X-100/PBS (PBST) for 10 min, blocked with 1% bovine serum albumin for 30 min, and stained with the indicated primary antibody for 3 h at room temperature. After washing three times with PBST, slides were incubated with secondary antibody for 2 h at room temperature. Fluorescence images were acquired using a Zeiss LSM 510 Meta confocal microscope (Carl Zeiss Inc., Oberkochen, Germany). The primary antibodies against LCN2, Snail, Twist, N-cadherin, and fibronectin were the same as those used for Western blotting. The secondary antibodies employed were Alexa Fluor 488-conjugated goat anti-mouse and Alexa Fluor 568-conjugated goat anti-rabbit (Invitrogen).

### Adenovirus-LCN2 generation

LCN2 cDNA was cloned into the pAd-track CMV vector, as described previously [22, 23]. Cells were infected with Ad-LCN2 or vector control (Ad-GFP) for 24 h, and subsequently examined by fluorescence microscopy and Western blotting.

### Animals

Xenograft models were prepared by injecting SCID mice with C33A-LCN2 cells using procedures similar to those described previously [24]. Mice were sacrificed for necropsy about 1 month after injection, and their livers and lungs were removed for tumor biopsy. Tumor volume was calculated using the equation, length × height × width. Formalin-fixed and paraffin-embedded tissues from SCID mice were evaluated by H & E staining and immunohistochemistry using a polyclonal antibody against LCN2 (Epitomics; #3639–1). All procedures were performed under sterile conditions in a laminar flow hood. Animal experiments were performed in accordance with the United States National Institutes of Health guidelines and the Chang-Gung Institutional Animal Care and Use Committee Guide for the Care and Use of Laboratory Animals.

### Human cervical cancer specimens

All samples of cervical cancer tissues with paired adjacent normal tissues for Western blot and immunohistochemistry analyses were from the Chang Gung Memorial Hospital. The study protocol was approved by the Medical Ethics and Human Clinical Trial Committee of the Chang Gung Memorial Hospital.

### Pathway enrichment analysis

Pathway enrichment analysis of a set of differentially expressed genes identified in an oligonucleotide microarray database of clinical specimens was performed using the GeneGo MetaCore analysis tool (GeneGo, St. Joseph, MI). Genes displaying differential expression greater than 1.5-fold were uploaded. A pathway map with a false discovery rate of < 0.01 was considered significant.

### ELISA of serum LCN2

The levels of serum LCN2 were measured using the Quantikine Human Lipocalin-2/NGAL Immunoassay kit (R & D Systems, Inc. Minneapolis, MN). The LCN2 protein standard or 50 μL of serum samples diluted up to 10-fold with the manufacturer-provided diluent in advance were transferred into a 96-well plate coated with a monoclonal antibody against LCN2. According to the manufacturer's instructions, the assay diluents and the conjugate reagent were added into every well step by step. After incubation, a substrate solution for the immunoperoxidase reaction was added to develop a color based on the amount of LCN2 antigen present in the samples. The reaction was terminated by adding a stop solution. Serum LCN2 concentration was quantitated by measuring the absorbance of each well at 450 nm. Serum LCN2 level was expressed as the ratio of standard curve (ng/dL) to adjust for changes in serum concentration.

### Statistical analysis

Data are expressed as mean values ± SEM of at least three experiments. Statistical analyses were performed using Student's *t* test and one-way analysis of variance (ANOVA). When appropriate, the Mann–Whitney *U* test or Fisher's exact test was used to compare the two groups, and a Kruskal–Wallis test or Pearson's χ^2^ test was used if more than two groups were compared. The relationship between the results of two different examinations was analyzed with Spearman's correlation test. Receiver operating characteristic (ROC) curve analysis was used to determine the cut-off points of the LCN2 histoscore in a univariate analysis. *P*-values < 0.05 were considered statistically significant. SPSS software was used for statistical analyses.

## SUPPLEMENTARY MATERIALS TABLES AND FIGURE


