# Supplementary material for: Overexpression of lipocalin 2 in human cervical cancer enhances tumor invasion
Source: Oncotarget. 2016 Jan 31;7(10):11113–26. doi: 10.18632/oncotarget.7096 (PMC4905461; doi:10.18632/oncotarget.7096)
Supplement: Supplementary file 1 [file oncotarget-07-11113-s001.pdf]

Overexpression of lipocalin 2 in human cervical cancer enhances tumor invasion

Supplementary Materials

Supplementary Table S1: Clinicopathologic correlations of LCN2 expression in patients with cervical cancer

Adenocarcinoma (AD)

Parameter	*n*	LCN2 histoscore	*P* value
**Age:**			
≤ 50	8	155 ± 84.515	0.205
> 50	20	196.5 ± 42.087	
**FIGO stage:**			
I b1	12	194.17	0.422
I b2	10	182	
II a	3	183.33	
II b	3	156.67	
**Histological grade:**			
Well	12	165.83 ± 75.131	0.591
Moderately	13	196.92 ± 41.309	
Poor	1	170	
**Histological grade:**			
Well	12	165.83 ± 75.131	0.220
Moderately/Poorly	14	195 ± 40.34	
**Tumor size(image):**			
≤ 4 cm	13	201.54 ± 72.439	0.056
> 4 cm	11	171.82 ± 42.147	
**Depth of penetration:**			
≤ 50%	8	160 ± 68.243	0.309
> 50%	18	193.89 ± 53.703	
**Lymph node involvement:**			
No	16	208.75 ± 37.394	0.009*
Yes	12	152.5 ± 67.974	

Adenosquamous carcinoma (ADSCC)

Parameter	*n*	LCN2 histoscore	*P* value
**Age:**			
≤ 50	5	150 ± 48.477	0.226
> 50	14	109.29 ± 53.989	
**FIGO stage:**			
I b1	11	116.36	0.422
I b2	2	95	
II a	2	155	
II b	4	125	
**Histological grade:**			
Well	1	130	0.941
Moderately	4	122.5 ± 29.861	
Poor	13	123.85 ± 61.582	

**Tumor size(image):**<br>≤ 4 cm<br>> 4 cm	7<br>7	150 ± 45.092<br>121.43 ± 61.582	0.441
**Depth of penetration:**<br>≤ 50%<br>> 50%	4<br>11	132.5 ± 84.212<br>128.18 ± 47.501	0.793
**Lymph node involvement:**<br>No<br>Yes	11<br>7	125.45 ± 52.795<br>121.43 ± 58.146	0.891

Supplementary Table S2: Pathway map enrichment analysis

#	Maps	Biological function	*P* value
1	Cytoskeleton remodeling	Cytoskeleton remodeling	4.07E–22
2	Regulation of epithelial-to-mesenchymal transition (EMT)	Cytoskeleton remodeling	1.60E–16
3	Clathrin-coated vesicle cycle	Transport	6.23E–16
4	IL-1 signaling pathway	Immune response	7.65E–14
5	JNK pathway	Signal transduction	3.03E–13
6	ECM remodeling	Cell adhesion	4.19E–13
7	Role of tetraspanins in the integrin- mediated cell adhesion	Cell adhesion	9.48E–12
8	TGF-beta-dependent induction of EMT via MAPK	Development	1.07E–11
9	Some pathways of EMT in cancer cells	Cell adhesion	1.33E–11
10	Integrin outside-in signaling	Cytoskeleton remodeling	4.68E–11

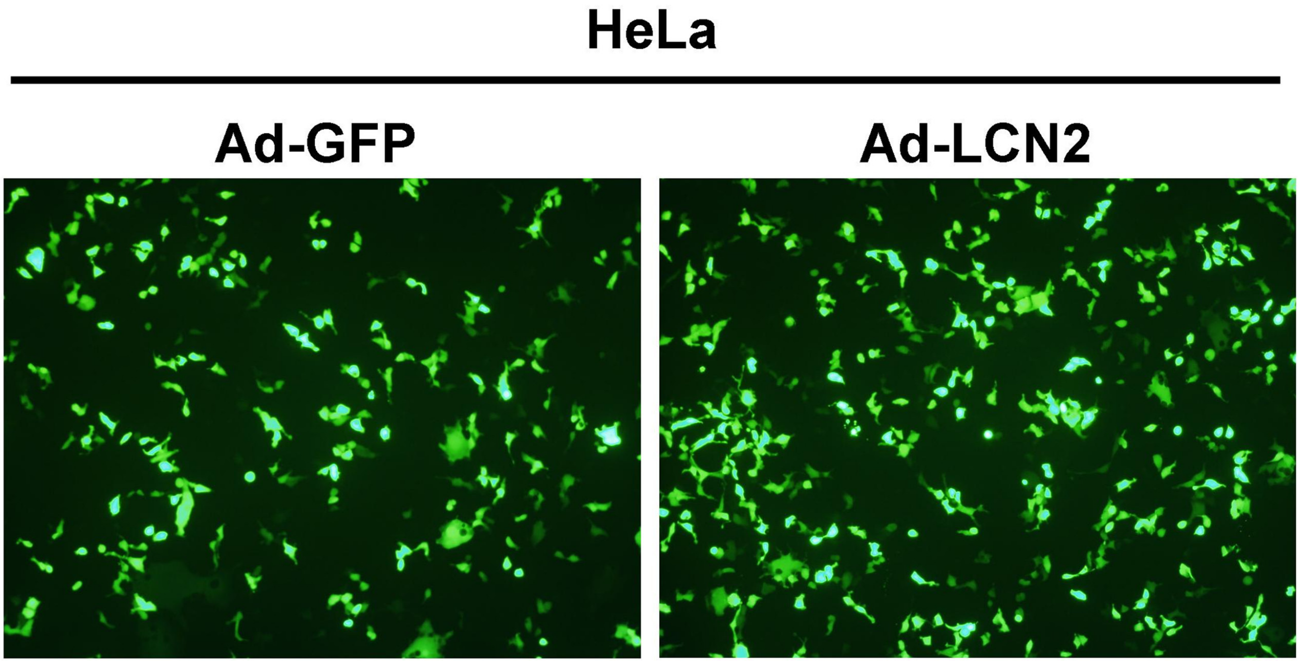

Supplementary Figure S1: Adenoviral-mediated overexpression of LCN2 in HeLa cells.
